# Characterization of muscle growth and sarcomere branching in the striated musculature of *C. elegans*

**DOI:** 10.1101/2024.08.30.610496

**Published:** 2024-08-30

**Authors:** A Fazyl, A Anbu, S Kollbaum, E Conklin, N Schroeder, AG Vidal-Gadea

**Affiliations:** 1 School of Biological Sciences, Illinois State University, Normal, IL; 2 Department of Crop Sciences, University of Illinois, Urbana, IL

## Abstract

Striated muscles are essential for locomotion and survival. Their function and structure are highly conserved across taxa. Muscles are highly plastic. Muscle growth can occur through several distinct processes including developmental, allometric, and hypertrophic growth. Additionally, pathological conditions like Duchenne Muscular Dystrophy (DMD) can lead to abnormal muscle growth. While human muscle studies have revealed complex structural adaptations such as sarcomere branching, these processes remain less explored in model organisms like *Caenorhabditis elegans*.

In this study, we present an anatomical characterization of muscle growth in *C. elegans* under various conditions that parallel those in mammalian systems. We examined developmental, allometric, and hypertrophic growth, as well as muscle atrophy in a DMD model, *dys-1(eg33)*. We find that *C. elegans* muscles undergo growth patterns similar to those observed in mammals, with region-specific increases in myocyte size, sarcomere number, and band widths under different conditions. Moreover, we report for the first time the presence of sarcomere branching and splitting in *C. elegans* muscles, phenomena previously described only in vertebrates and Drosophila.

We further report that sarcomere branching is modulated by environmental conditions and pathological states, with increased branching in worms raised swimming and reduced branching in dystrophic muscles. These findings provide new insights into the mechanisms of muscle adaptation and highlight the potential of *C. elegans* as a model for studying muscle pathologies like DMD, particularly during periods of rapid growth.

## INTRODUCTION

Striated muscles are highly specialized tissues that play a crucial role in posture, the production of movement, and overall metabolic health. In mammals, striated muscles comprise long, multinucleated cells known as myofibers. Within them, sarcomeres function as the basic contractile element of the muscle (Frontera and Ochala, 2015). Sarcomeres and muscles in general are highly conserved across animal taxa (Mendoza et al., 2023).

One of the key features of muscles is their ability to adapt to changes in their output and metabolic demands. Multiple dynamic processes ensure that these tissues are constantly finetuned to their everchanging environmental and metabolic surroundings. The different mechanisms governing positive (hypertrophy) or negative (atrophy) changes in muscle size are among the clearest examples of these processes (Sartori et al., 2021).

**Figure 1.**
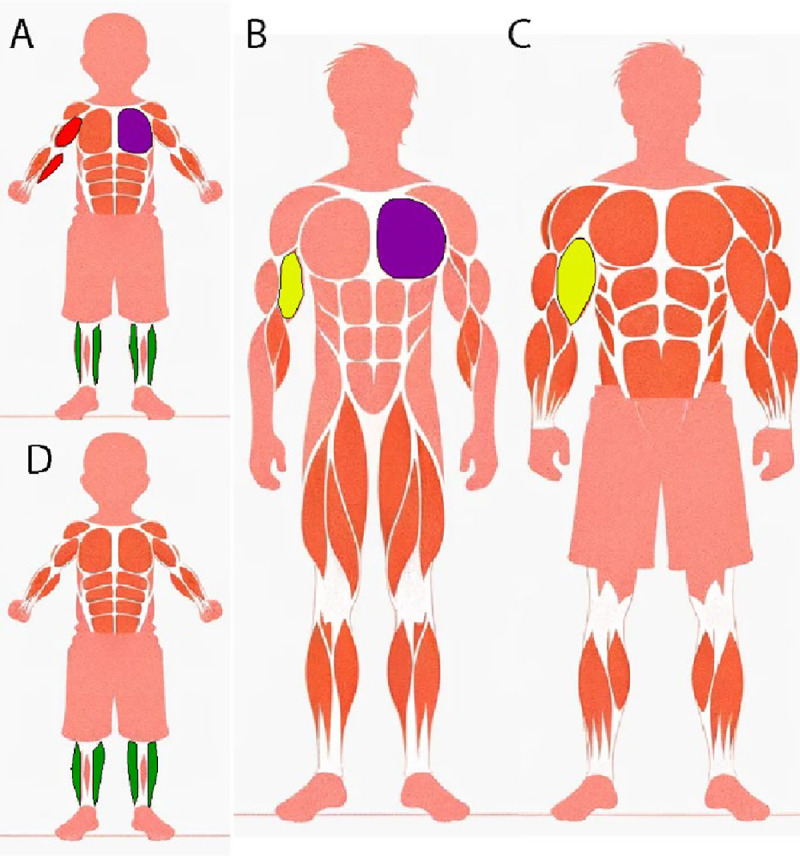
Visualization of muscle growth types and atrophy across different developmental stages and dystrophic conditions in human. **A.** Schematic of a healthy child displaying various muscle growth types. Red regions indicate allometric growth, where muscle size scales relative to the body part. In this example, it is the arm muscles of different size. The magenta area highlights developmental growth, characterized by the growth of muscles during early life (to be compared with B). **B.** Schematic of a healthy adult showing the distribution of muscle growth types. The magenta region on the chest signifies developmental growth from childhood, while the yellow areas in the arms represent hypertrophic growth, where muscles increase in size due to resistance training (to be compared with C). **C.** Another schematic of a healthy adult male focusing on hypertrophic growth, particularly in the arm (yellow regions), reflecting the muscle enlargement due to the training. **D.** Schematic of a child with DMD. The calves are highlighted in green, indicating hypertrophic growth compared to healthy child’s calves in A. This hypertrophic growth is characteristic of DMD patients due to the compensatory increase in muscle size despite overall muscle weakness.

Muscle growth can occur through several overlapping but distinct mechanisms, including developmental growth, allometric growth, hypertrophic growth, and pathological growth such as that observed in muscular dystrophies (Figure 1). Developmental growth is a genetically programmed process that occurs during early life, leading to the formation of mature myofibers from precursor cells. This process is governed by a tightly regulated network of signaling pathways that control cell proliferation, differentiation, and fusion (Biressi & Rando, 2010). In contrast, allometric growth is characterized by the scaling of muscle size relative to the overall growth of the organism, involving intricate interactions between genetic factors and environmental stimuli (White et al., 2010). Developmental and allometric growth are largely the result of hardwired genetic programs. However, muscles are also able to grow in response to external stimuli.

Hypertrophic growth is a type of activity-dependent muscle growth that occurs in response to increased mechanical load, such as during resistance exercise, and is characterized by an increase in the size of existing muscle fibers rather than the formation of new ones (as seen during development). This process is mediated by key signaling pathways, including the mTOR pathway, which promotes protein synthesis and muscle fiber enlargement (Glass, 2005). Hypertrophic growth can result from increases in the contractile machinery within the muscle (myofibrillar hypertrophy), or from increases in the sarcoplasm and non-contractile elements of muscles (sarcoplasmic hypertrophy, Schoenfeld, 2010).

Pathological conditions can also result in muscle growth. For example, the muscles of Duchenne Muscular Dystrophy (DMD) patients can display pseudo-hypertrophy where muscle growth results from the accumulation of non-functional tissues associated with the degeneration of these cells (Hoffman, Brown, & Kunkel, 1987).

These different types of muscle growth might occur in isolation or might be found in combination within a single muscle. A thorough understanding of how the various forms of muscle growth alter muscle structure and function is essential from a basic biology standpoint, but also crucial from a clinical one (Schiaffino & Reggiani, 2011). Diseases like DMD are known to affect some muscles more severely than others. It is likely that at least part of this differential muscle susceptibility to DMD might be due to differences in the interaction between the disease and the different growth states undergone by these muscles.

Much remains to be learned about striated muscle structure, function, and plasticity. For example, recent work using human biopsies discovered that myofibrils in human muscles do not exist as independent parallel fibers but are interconnected by branching processes which effectively link all myofibrils into a force-producing network (Willingham et al; 2020). The discovery of myofibril branching represents a significant departure from the traditional view of their organization and suggests a new layer of complexity in muscle adaptation and pathology.

This is particularly true for the pathophysiology Duchenne muscular dystrophy (DMD), a common and lethal muscular disease caused by mutations resulting in the loss of the dystrophin protein. Despite much research, there is limited knowledge about how the pathophysiology of the disease is altered by the different growth states of afflicted muscles. This information is of crucial importance since patients are usually diagnosed during infancy when they are undergoing their most diverse and intense period of muscle growth. Therefore, understanding the pathophysiology of DMD requires not simply modeling the disease genetically, but also the modeling of the different growth states of the musculature it afflicts.

**Figure 2.**
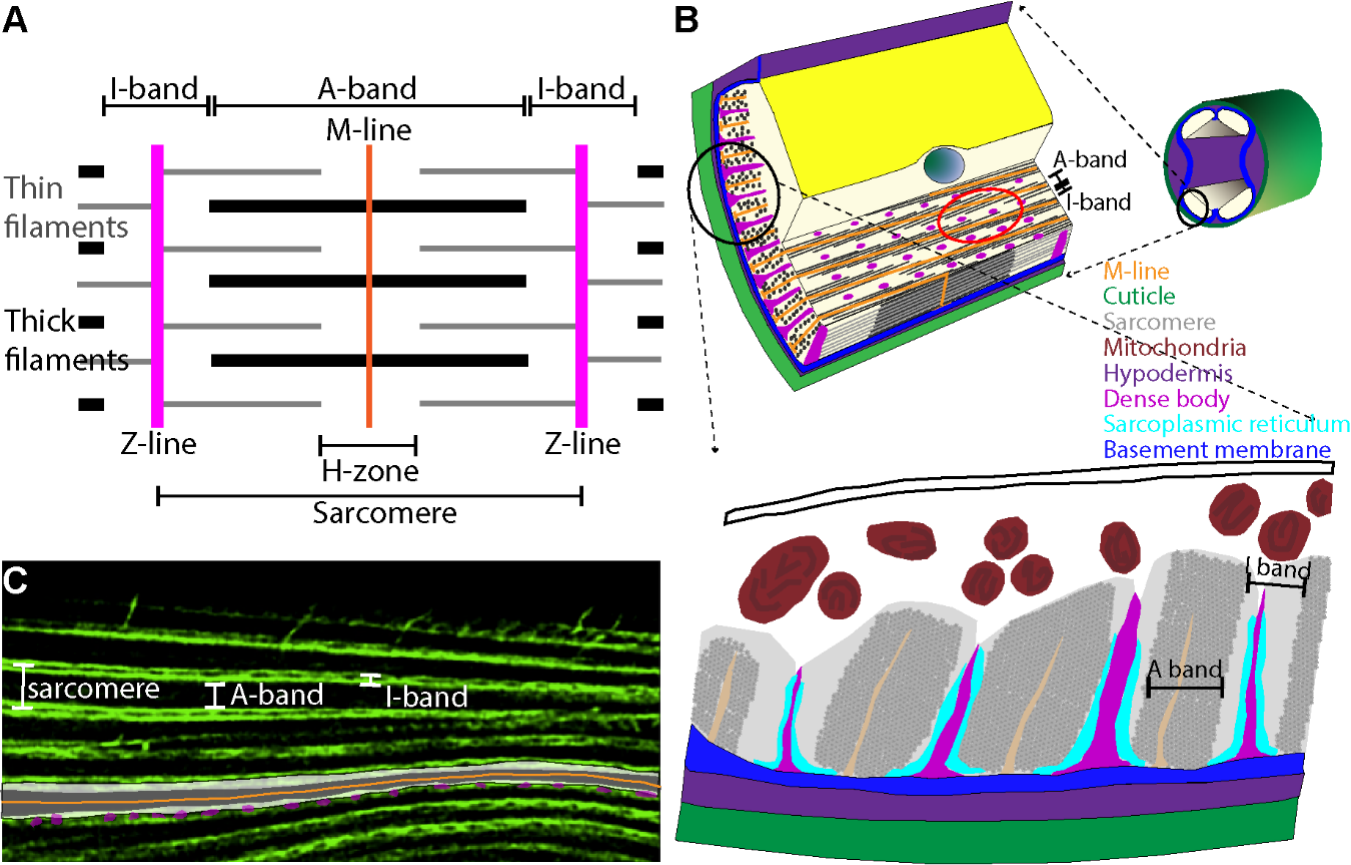
Schematics and image of sarcomere structure in *C. elegans* striated muscles. **A.** Diagram representing the sarcomere, a contractile unit of striated muscles. The sarcomere is bordered by Z-lines (magenta), which anchor the thin filaments (grey lines) composed of actin. The A-band, which includes the M-line (orange) at its center, represents the region containing thick filaments (black lines) composed of myosin. The I-band, adjacent to the Z-lines, consists of thin filaments only. The H-zone within the A-band represents the region containing thick filaments that do not overlap with thin filaments. **B.** Cross-sectional view of *C. elegans* body wall muscle showing the arrangement of sarcomeres within the myocyte. The schematic illustrates key muscle components. The enlarged section emphasizes the A-band and I-band, reflecting the structural organization of sarcomere. **C.** Confocal image of *C. elegans* striated muscle, stained with phalloidin to visualize F-actin filaments. The image shows a sarcomere with clearly identifiable A-bands and I-bands. The labeling corresponds to the spatial organization of contractile elements within myocyte illustrated in the schematic in A and B. Figure modeled after Gieseler et al., 2018.

For decades the nematode *Caenorhabditis elegans* (*C. elegans*) has been used to model striated muscles and the disorders that afflict them (Moerman and William, 2006; Gieseler et al., 2018; Ellwood et al., 2021). The striated musculature of *C. elegans* comprises 95 mononucleated myocytes arranged in four quadrants spanning the length of the animal. Aside from some structural differences, *C. elegans* muscles closely resemble mammalian muscles in structure and function (Figure 2, Gieseler et al., 2018).

Our lab uses *C. elegans* to model DMD genetically and phenotypically (Beron et al., 2015). Recent work in our lab showed that *C. elegans* raised burrowing through solid agar media undergo activity-induced hypertrophic growth, and DMD-related pseudohypertrophic growth (Hughes et al., 2018). Here we perform an anatomical characterization of different types of muscle growth in *C. elegans* that parallel mammalian muscle growth. Furthermore, paralleling recent discoveries in human striated muscles we show that *C. elegans* body wall muscles exhibit sarcomere branching and splitting and find that the extent of sarcomere branching is plastic. This work highlights the usefulness of *C. elegans* as a model for studying muscle biology but also opens new avenues for investigating the molecular mechanisms underlying sarcomere branching in response to different growth conditions and pathologies.

## RESULTS

To study myocyte growth we compared *C. elegans* raised under different conditions. We compared anterior, medial, and posterior myocytes in day 1 adults raised on solid agar plates (crawling). This allowed us to compare myocytes that reach different sizes through allometric growth. To then compare myocyte growth through development we compared myocytes from day 1 and day 5 adults raised crawling on agar plates. This allowed us to focus on developmental growth without the confounding variable of molting which animals undergo during larval growth. To make investigate the effect of activity-dependent (hypertrophic) growth in muscles we compared day 1 adults that were raised crawling to those raised swimming (in liquid media). Lastly, to look at the effect of Duchenne muscular dystrophy on myocytes we compared healthy and dystrophic, *dys-1(eg33)*, day 5 adults raised on agar plates.

We performed a Pearson product moment correlation analysis of between 26 myocytes ranging in area between 561μm^2^ and 1,738μm^2^ comparing their number of sarcomeres, their average sarcomere, A-band, and I-band widths (Table 1). We found that all these parameters were significantly correlated with myocyte size, with sarcomere number, A-band width and I-band width having the strongest and most significant coefficients. We therefore set out to compare sarcomere number and A-band and I-band widths for myocytes under different growth regimens.

### Allometric growth in *C. elegans* striated muscles

**Figure 3.**
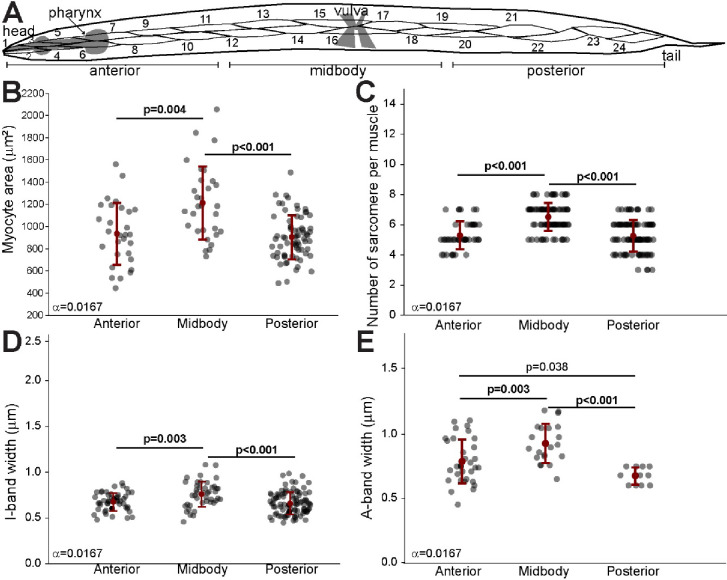
Allometric growth *C. elegans* striated muscles across different body regions. **A.** Schematic of an adult C. elegans showing anterior, midbody, and posterior regions, which correspond to different muscle groups analyzed in this study. Numbers represent individual myocytes in each region. **B.** Quantification of myocyte area across the anterior, midbody, and posterior regions. Each point represents individual myocyte. **C.** Number of sarcomeres per myocyte in the anterior, midbody, and posterior regions. Each point represents the average sarcomere number per myocyte. **D.** Measurement of I-band width across different body regions. I-band width shows the length of thin filaments in the sarcomere where actin filaments are not overlapped by myosin. Each point represents average I-band width per myocyte. **E.** Measurement of A-band width across different body regions. A-band width corresponds to the length of the thick filaments where myosin and actin filaments overlap. Each point represents average A-band width per myocyte. Error bars = SEM, Bonferroni corrected alpha displayed by each comparison. Significant p values displayed in bold.

To investigate the patterns of allometric growth in *C. elegans* body wall muscles, we analyzed the myocyte area, sarcomere number, and the widths of sarcomere bands (A-band and I-band) across different body regions, specifically the anterior, mid-body, and posterior segments (Figure 3A). As previously described (Gieseler et al., 2018), we found regional variation in muscle growth, with the mid-body myocytes exhibiting a significantly larger area compared to both the anterior and posterior regions (Figure 3B). Consistent with the increase in myocyte area, we observed that the number of sarcomeres within the myocytes followed a similar trend, with the mid-body containing a greater number of sarcomeres compared to the anterior and posterior regions (Figure 3C). Additionally, we measured the widths of the I-band and A-band, which are key components of the sarcomere. The I-bands were significantly wider in the mid-body compared to both the anterior and posterior regions (Figure 3D). The A-band also exhibited regional differences. We found that the mid-body region had a significantly wider A-band compared to both the anterior and posterior regions (Figure 3E). Overall, these results characterize allometric growth of body wall muscles in *C. elegans* as being accompanied by a general increase in the size and number of sarcomeres.

### Developmental growth in *C. elegans* striated muscles

**Figure 4.**
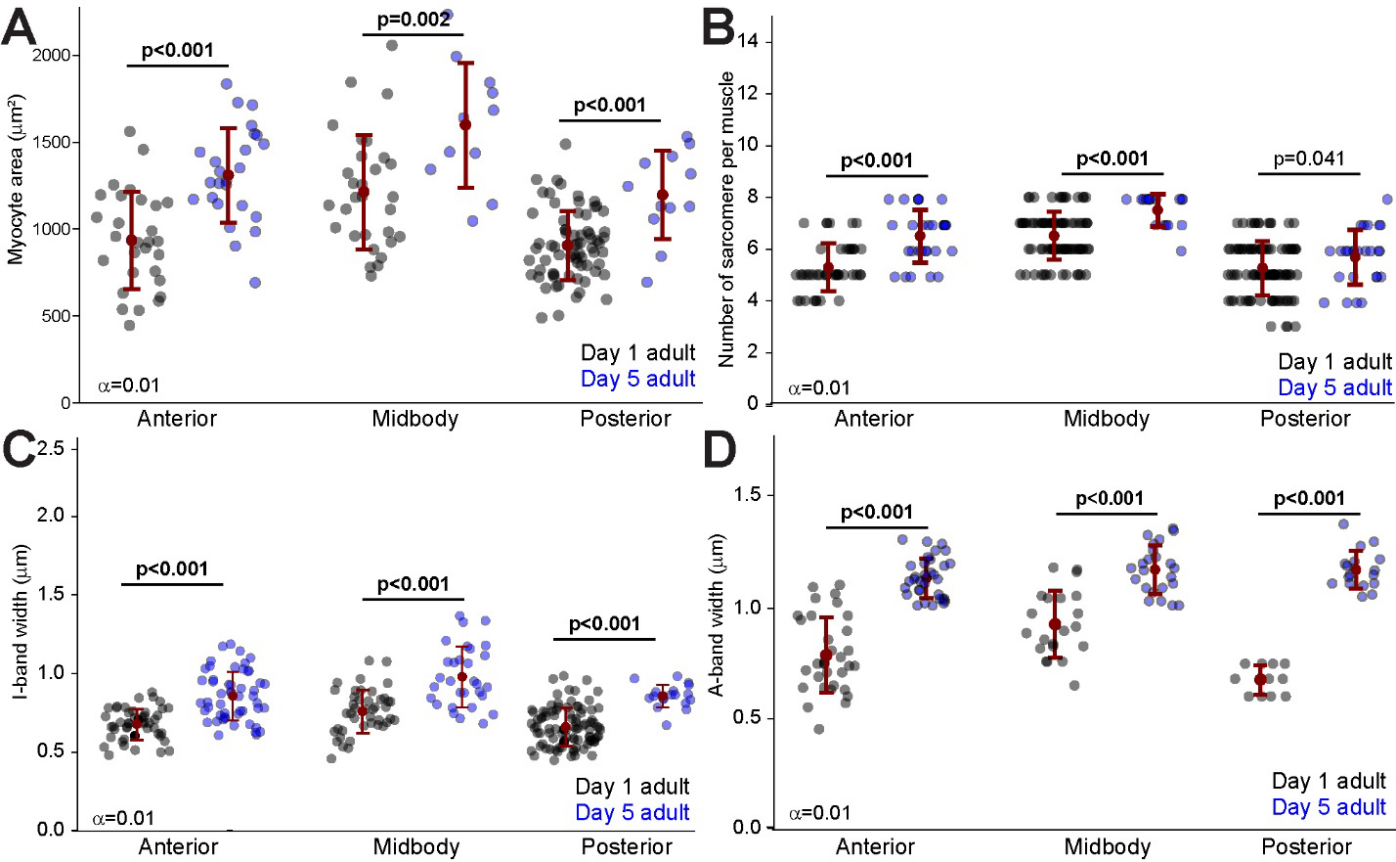
Developmental growth of *C. elegans* striated muscles from day 1 to day 5 of adulthood. **A.** Quantification of myocyte area in the anterior, midbody, and posterior regions of *C. elegans* at day 1 and day 5 of adulthood. Each point represents an individual myocyte. **B.** Number of sarcomeres per myocyte in the anterior, midbody, and posterior regions at day 1 and day 5 of adulthood. Each point represents the sarcomere number per myocyte. **C.** Measurement of I-band width across different body regions at day 1 and day 5 of adulthood. I-band width reflects the length of thin filaments in the sarcomere where actin filaments are not overlapped by myosin. Each point represents average I-band width in a myocyte. **D.** Measurement of A-band width across different body regions at day 1 and day 5 of adulthood. A-band width corresponds to the length of the thick filaments where myosin and actin filaments overlap. Each point represents average A-band width in a myocyte. Error bars = SEM, Bonferroni corrected alpha displayed by each comparison. Significant p values displayed in bold.

Development in *C. elegans* is characterized by periods of continuous growth interrupted by four molting events that are followed by periods of rapid growth. Once they reach adulthood, *C. elegans* worms continue to grow up to 30% over their first week of adulthood. To understand the developmental growth patterns in *C. elegans* striated muscles without the confounding effect of the molting process we measured and compared the muscles of day 1 and day 5 adult worms. We found a general increase in myocyte size across the anterior and medial body regions (Figure 4A). Like our findings for allometric growth, increased myocyte was accompanied by an increase in the number and size of the sarcomeres (Figure 4B). Additionally, the I-band width, which is indicative of the sarcomere’s thin filament region, was significantly wider in day 5 adults compared to day 1 adults across all body regions (Figure 4C). The same trend was observed for the A-band width, with all three body regions showing significant increases in day 5 adults compared to day 1 adults (Figure 4D). These results are consistent with the idea that developmental growth in *C. elegans* involves significant increases in myocyte size, sarcomere number (with the exception of the posterior region), and both I-band and A-band widths as the animals grow from day 1 to day 5 of adulthood.

### Hypertrophic growth in *C. elegans* striated muscles

Under laboratory conditions *C. elegans* are routinely raised crawling on the surface of solid agar plates, or swimming in liquid media (Stiernagle, 2006). Previous work showed that swimming and crawling are distinct locomotor behaviors which place differential demands on the body wall musculature (Pierce-Shimomura et al., 2008; Vidal-Gadea et al., 2011; Beron et al., 2015; Fazyl et al., 2024). To explore how the differential demands of these behaviors alter muscle growth we compared the body wall muscles of worms grown on crawling on solid agar or raised in a liquid nematode growth media (NGM).

**Figure 5.**
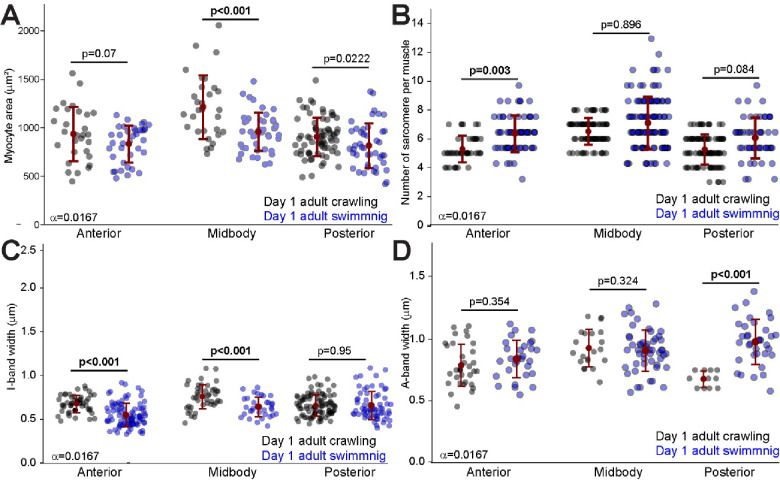
Hypertrophic growth in *C. elegans* striated muscles under different environmental conditions (crawling vs. swimming). **A.** Quantification of myocyte area in the anterior, midbody, and posterior regions of *C. elegans* grown in crawling (hard NGM agar) versus swimming (liquid NGM) environments. Each point represents an individual myocyte. **B.** Number of sarcomeres per myocyte in the anterior, midbody, and posterior regions of *C. elegans* in crawling versus swimming environments. Each point represents the sarcomere number per myocyte. **C.** Measurement of I-band width across different body regions in crawling versus swimming environments. I-band width reflects the length of thin filaments in the sarcomere where actin filaments are not overlapped by myosin. Each point represents average I-band width in a myocyte. **D.** Measurement of A-band width across different body regions in crawling versus swimming environments. A-band width corresponds to the length of the thick filaments where myosin and actin filaments overlap. Each point represents average A-band width in a myocyte. Error bars = SEM, Bonferroni corrected alpha displayed by each comparison. Significant p values displayed in bold.

Midbody myocytes from crawling worms were significantly larger than their swimming counterparts (Figure 5A). Other metrics did not parallel this observation. We found that anterior myocytes from swimming worms had a higher number of sarcomeres compared to crawling worms (Figure 5B). Within sarcomeres, the anterior and medial myocytes of crawling worms displayed greater I-band width than in swimming worms (Figure 5C), however A-band width was greater for the posterior myocytes of swimming worms (Figure 5D). These findings indicate that activity-dependent (hypertrophic) growth in *C. elegans* varies in terms of which muscles and which parameters are affected.

### Muscle atrophy in *C. elegans* dystrophic model

**Figure 6.**
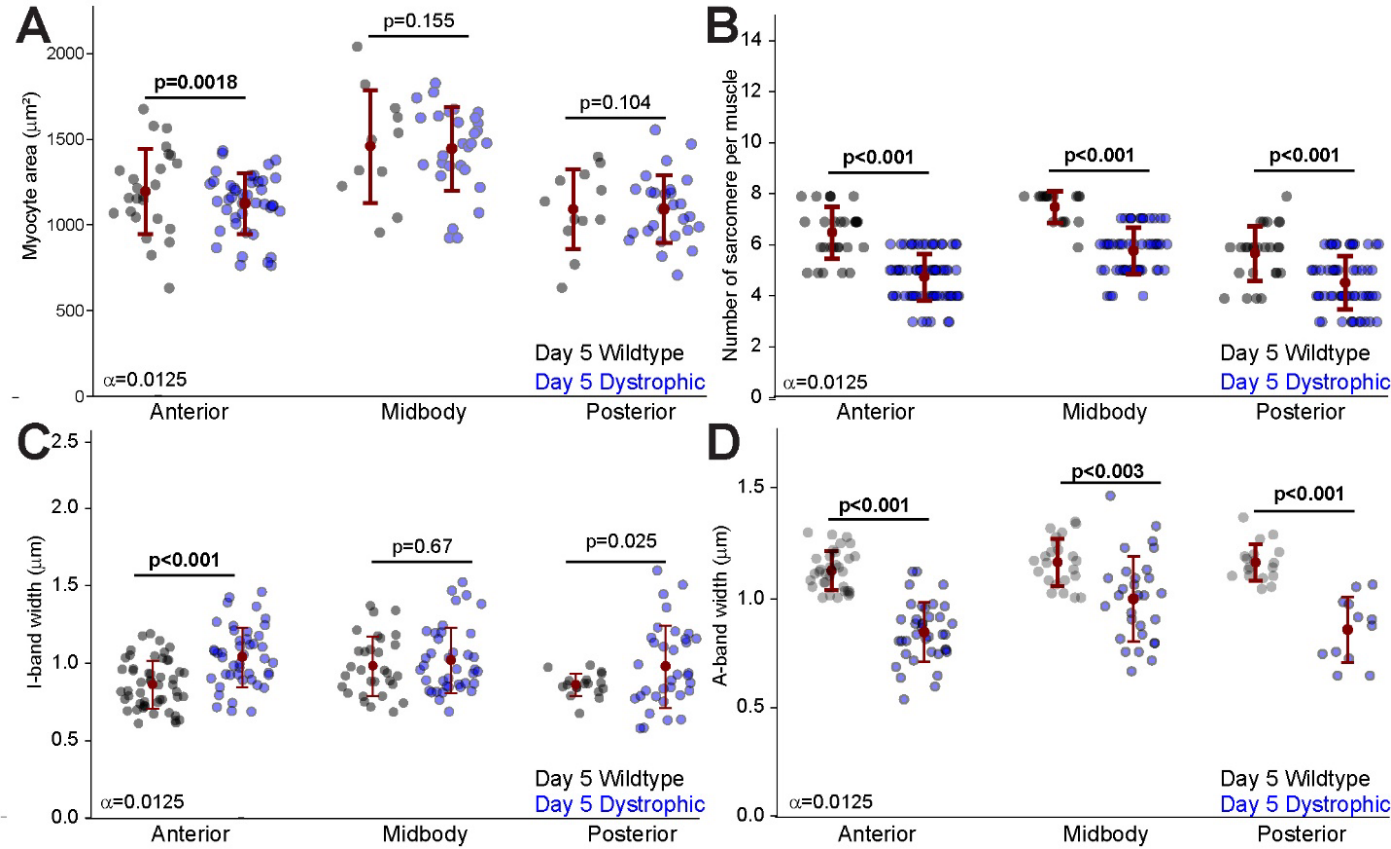
Muscle atrophy in dystrophic *C. elegans* compared to wild-type at day 5 of adulthood. **A.** Quantification of myocyte area in the anterior, midbody, and posterior regions of wild-type and dystrophic *C. elegans* at day 5 of adulthood. Each point represents an individual myocyte. **B.** Number of sarcomeres per myocyte in the anterior, midbody, and posterior regions of wild-type and dystrophic *C. elegans* at day 5 of adulthood. Each point represents the sarcomere number per myocyte. **C.** Measurement of I-band width across different body regions in wild-type and dystrophic *C. elegans* at day 5 of adulthood. I-band width reflects the length of thin filaments in the sarcomere where actin filaments are not overlapped by myosin. Each point represents average I-band width in a myocyte. **D.** Measurement of A-band width across different body regions in wild-type and dystrophic *C. elegans* at day 5 of adulthood. A-band width corresponds to the length of the thick filaments where myosin and actin filaments overlap. Each point represents average A-band width in a myocyte. Error bars = SEM, Bonferroni corrected alpha displayed by each comparison. Significant p values displayed in bold.

For over twenty years *C. elegans* has been used to model Duchenne muscular dystrophy. Most characterizations of dystrophic (*dys-1*) worms have focused on myocyte or muscle longevity and output (Ellwood et al., 2021). In a previous study we showed that burrowing through solid agar was able to trigger myocyte growth while also a decrease in sarcomere width in dystrophic animals (Hughes et al., 2019). We compared the myocytes of day-5 dystrophic *dys-1(eg33)* worms raised crawling to healthy day-5 animals and found a decrease in anterior myocyte size in dystrophic animals (Figure 6A). Interestingly, we also observed a significant reduction in the number of sarcomeres across the entire body wall musculature (Figure 6B). We found that while the I-band width of anterior dystrophic myocytes was greater than their healthy counterparts (Figure 6C), A-band widths were significantly smaller for all the myocytes of *dys-1* animals (Figure 6D).

### Sarcomere branching and splitting in *C. elegans* muscles

**Figure 7.**
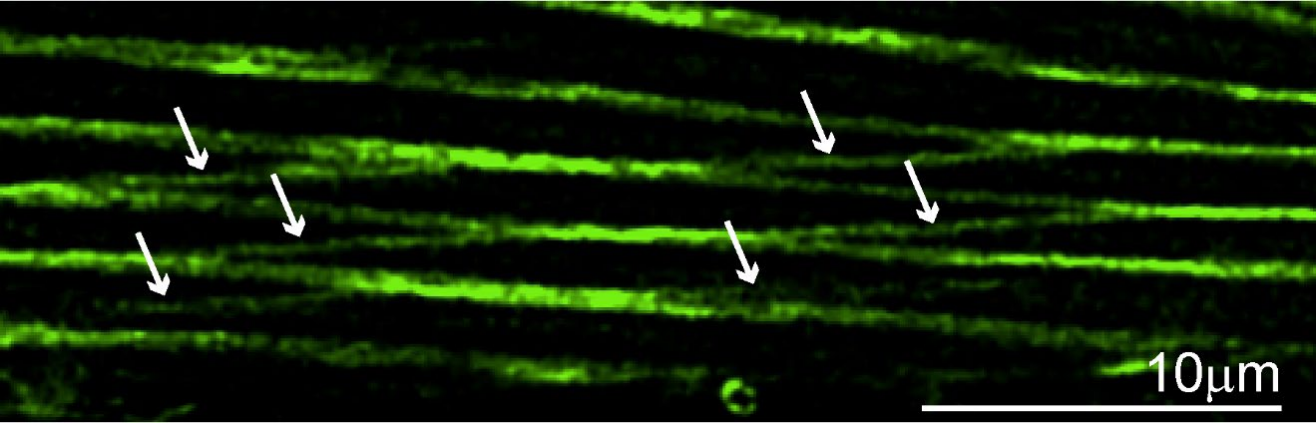
Sarcomere branching in the midbody region of a swimming worm. Confocal image of the middle region of body wall muscle from a swimming *C. elegans*, stained with phalloidin to visualize F-actin filaments. The image shows multiple distinct sarcomere branching events. Arrows indicate the sarcomere branching.

**Figure 8.**
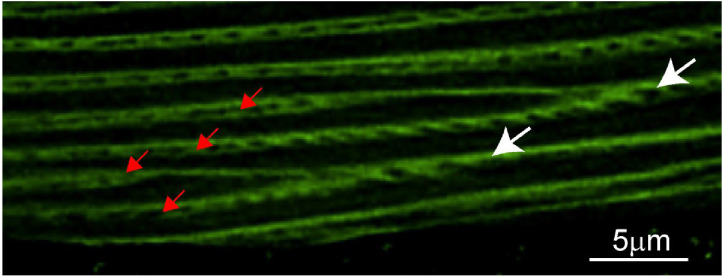
Sarcomere splitting in the anterior body wall muscle of a swimming *C. elegans*. Confocal image of an anterior body wall muscle from a swimming *C. elegans* stained with phalloidin to visualize F-actin filaments, showing multiple sarcomere splitting events. White arrows indicate two parental (original) sarcomeres, while red arrows indicate four resulting sarcomeres formed through the splitting process.

**Figure 9.**
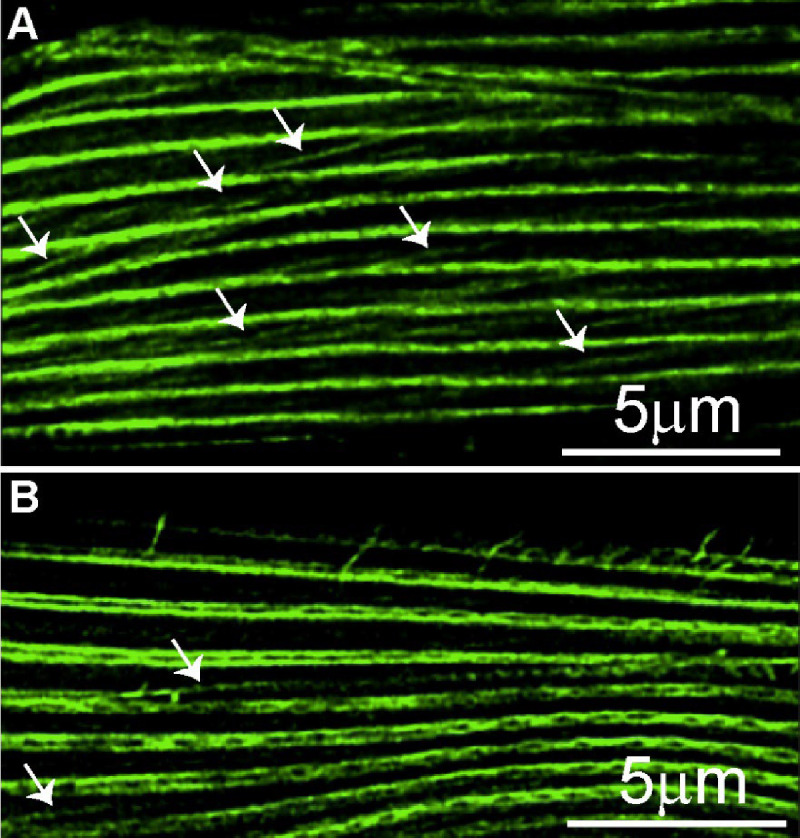
Variability in sarcomere branching in the anterior region of body wall muscle from a crawling *C. elegans*. **A.** Confocal image of muscle displaying a greater amount of sarcomere branching throughout the myocyte, with branches showing similar lengths. **B.** Confocal image of muscle displaying reduced sarcomere branching. Arrows indicate the sarcomere branches.

Recent work using human and Drosophila myocytes revealed the presence of myofibrillar networks where the forces generated by individual sarcomeres were not just transferred within a single myofibril but were also laterally transferred to other myofibrils through actin branches connecting them (Højfeldt et al., 2023; Ajayi et al., 2022; Willingham et al., 2020). These myofibrillar networks are believed to allow all the sarcomeres within a myofiber to act in concert. Combining phalloidin staining with high resolution (Leica Lightning) confocal imaging of *C. elegans* muscles, we noticed a phenomenon that closely resembles the mammalian myofibrillar networks. Specifically, we found that body wall muscles displayed actin connections between adjacent sarcomeres (which we tentatively termed sarcomere branching, Figure 7). We also found many instances where a single sarcomere branched to give rise to two independent sarcomeres (we termed this sarcomere splitting, Figure 8). In humans and fruit flies, myofibrillar branching and splitting I muscle specific, with some myocytes displaying extensive branching while others displayed none (Ajayi et al., 2022). We similarly found that while some myocytes displayed extensive branching, others lacked it entirely (Figure 9). We therefore set out to compare this phenomenon between different muscles and under different growth conditions.

### Sarcomere branching under different growth conditions

**Figure 10.**
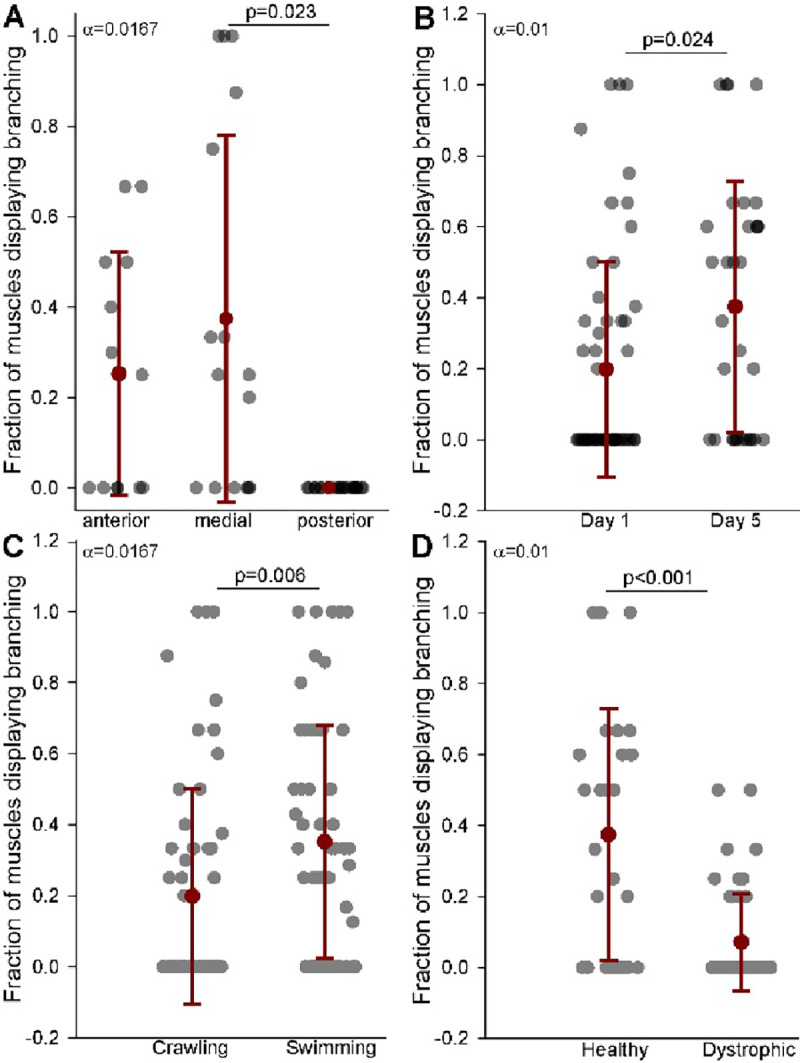
Fraction of muscles displaying sarcomere branching under various conditions. **A.** Fraction of muscles displaying sarcomere branching across anterior, medial, and posterior regions of the body. **B.** Comparison of sarcomere branching in muscles at day 1 and day 5 of adulthood. **C.** Fraction of muscles displaying sarcomere branching in *C. elegans* grown under crawling versus swimming conditions. **D.** Fraction of muscles displaying sarcomere branching in healthy versus dystrophic *C. elegans*. Error bars = SEM, Bonferroni corrected alpha displayed by each comparison. Significant p values displayed in bold.

**Figure 11.**
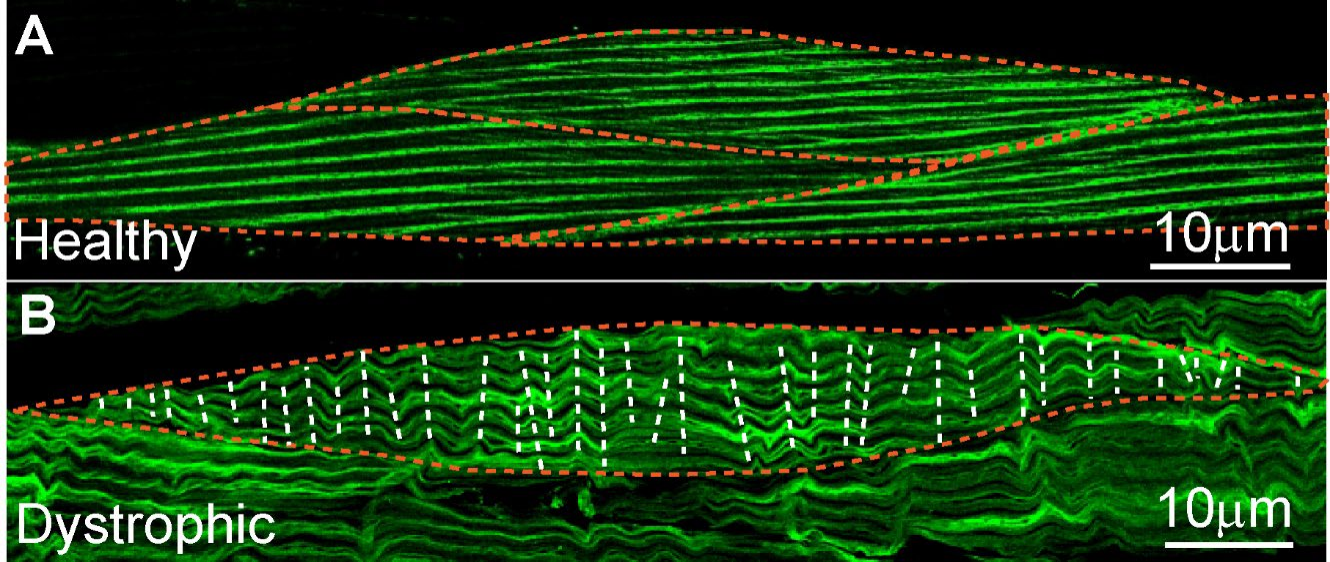
Comparison of myocyte structure between healthy and dystrophic *C. elegans*. **A.** Confocal image of healthy muscle displaying well-organized sarcomeres with consistent alignment across the myocyte. **B.** Confocal image of dystrophic muscle from *dys-1(eg33)* mutant showing significant structural abnormalities, including irregular and wavy sarcomeres. The dashed lines highlight the disruption in sarcomere organization and structure.

We quantified the fraction of muscles displaying sarcomere branching across body regions in day 1 adult wildtype worms and found no significant difference between anterior, medial, or posterior myocytes (Figure 10A). Similarly, we found no differences when we compared myocytes between day 1 and day 5 adults (Figure 10B). No significant difference was detected. To determine whether environmental conditions influence sarcomere branching, we compared *C. elegans* grown in crawling versus swimming environments and found that a greater fraction of myocytes from animals raised swimming displayed sarcomere branching (Figure 10C). Conversely, we observed sarcomere branching in a significantly lower fraction of dystrophic myocytes (Figure 10D). The absence of sarcomere branching in dystrophic muscles usually coincided with the presence of wavy sarcomeres (Figure 11).

## DISCUSSION

We used standard anatomical techniques to present a direct comparison between *C. elegans* striated myocytes undergoing different types of growth and show a parallel to established patterns of muscle growth in humans. This included developmental and allometric myocyte growth in *C. elegans* which was characterized by a global increase in myocyte metrics including increased sarcomere number and size as also seen in humans and other mammals (Figures 3 and 4; Goldspink, 1968, White et al., 2010).

In humans, activity-dependent (hypertrophic) growth involves localized and specific alterations to myocyte structure and (Schiaffino & Reggiani, 2011). As in humans we found that activity-dependent muscle growth (Hypertrophy) affected different muscles in different ways. For example, we found that medial myocytes from worms raised crawling were significantly larger than their swimming counterparts, while anterior myocytes from swimming worms had significantly more sarcomeres. Similarly, while the sarcomeres of anterior and medial myocytes of worms raised crawling had greater I-band widths, the sarcomeres of their posterior myocytes had reduced A-band widths (Figure 5).

As in humans we found that activity-dependent muscle growth (Hypertrophy) affected different muscles in different ways. For example, we found that medial myocytes from worms raised crawling were significantly larger than those from their swimming counterparts, while anterior myocytes from swimming worms had significantly more sarcomeres. Similarly, while the sarcomeres of anterior and medial myocytes of worms raised crawling had greater I-band widths, the sarcomeres of their posterior myocytes had reduced A-band widths (Figure 5).

For decades *C. elegans* has been fruitfully used to model Duchenne muscular dystrophy. We found several differences between the myocytes from healthy and dystrophic day-5 adult worms. As expected, we found a reduction in the size of some dystrophic muscles (Figure 6A). However, we also found a significant reduction in the number of sarcomeres per myocyte (Figure 6B) and in the width of their A-band (Figure 6D). Both a reduction in sarcomeres and in the length of the thick filaments (which determine the length of the A-band) would be consistent with a reduction in force production consistent with known dystrophic phenotypes (Wokke et al., 2014). However, it remains unclear if these changes are compensatory or part of the dystrophic pathophysiology).

Myofibril networks were recently described in striated myocytes of humans and Drosophila (Ajayi et al., 2022). We provide the first description of sarcomere branching and splitting in the body wall muscles of *C. elegans* (Figures 7–9). As in humans, we found that branching varied significantly between muscles. Comparison of the fraction of myocytes displaying sarcomere branching between animals raised swimming or crawling suggests that sarcomere branching is not hardwired but can be modulated by experience (Figure 10C). While swimming animals had a significantly greater fraction of myocytes displaying branching, we found that dystrophic myocytes displayed the opposite trend (Figure 10D). Loss of sarcomere or myofibrillar network integrity has not been previously reported in dystrophic muscles. One characteristic of degenerating dystrophic myocytes in *C. elegans* is the appearance of wavy sarcomeres as the disease progresses. Future studies could investigate if the loss of these branches causes a redistribution of the lateral tension experience by sarcomeres in a muscle resulting in this wave like pattern we observe (Figure 11). If this proves to be the case, loss of sarcomere branching in DMD would be a previously undescribed pathophysiological step leading to the weakness observed in dystrophic muscles.

The striated musculature of *C. elegans* continues to be a useful system to model human muscles and disease. There are several differences between nematode and mammalian muscles. Unlike mammalian muscles, the 95 myocytes comprising *C. elegans* striated musculature do not fuse and thus remain mononucleated. Worms lack satellite cells and therefore cannot readily replace dead myocytes. While these differences are important and caution immediate or direct comparisons, their flip side is that they are often advantageous as they permit the investigation of muscle structure and function at the single myocyte level in identifiable cells. Our present work highlights the utility of *C. elegans* for the study of muscle growth in health and disease. This is of particular importance in the context of muscular dystrophies such as Duchenne where patients are usually diagnosed during infancy, a period during which they are undergoing rapid muscle growth. Understanding and faithfully modeling different types of muscle growth will be of crucial importance when studying the pathophysiology of Duchenne and other muscular disorders.

## METHODS

### Strains

*C. elegans* strains used in this study included wild-type N2 and dystrophic BZ33 *dys-1(eg33)* mutant. Worms were grown under standard conditions as described previously (Brenner, 1974).

### Experimental design

The study examined muscle growth under three conditions: hypertrophic, developmental, and allometric growth, as well as muscle atrophy in dystrophic *dys-1 (eg33)* worms.

### Hypertrophic growth

For hypertrophic growth experiments, worms were cultured in two distinct environments: hard NGM agar for crawling and liquid NGM agar for swimming. Worms were bleached to synchronize eggs, which were then placed in either liquid or hard NGM agar. Worms were allowed to grow to the day 1 adult stage in the second generation before further analysis.

### Developmental growth

Developmental growth was studied by growing worms on hard NGM agar plates. Eggs obtained through bleaching were placed on the plates and allowed to develop until the worms reached day 1 and day 5 of adulthood.

### Allometric growth

For allometric growth analysis, different parts of the worm (head, midbody, tail) were studied. Worms were grown on hard NGM agar plates from eggs to the day 1 adulthood.

### Muscle atrophy

To study muscle atrophy, *dys-1 (eg33)* worms were grown on hard NGM agar plates under standard crawling conditions. Eggs were placed on the plates following bleaching, and worms were allowed to grow to the day 5 adult stage.

### Fixation and staining

Worms at the desired stages (day 1 and day 5 adults) were collected from the plates or liquid media and fixed in 4% paraformaldehyde in phosphate-buffered saline for 15mins at room temperature. Fixed worms were then subjected to freeze-cracking to permeabilize the cuticle. Following permeabilization, samples were stained with iFluor 488-conjugated phalloidin for overnight to visualize F-actin filaments.

### Imaging and analysis

Stained worms were imaged using a Leica SP8 confocal microscope. Confocal images were captured using Lightning feature to obtain high-resolution images of muscle structures. Images were processed and analyzed using Leica Application Suite X (LasX) software and ImageJ. Quantitative analysis included measurements of myocyte size, sarcomere size, A-band, and I-band, and morphology across different regions (head, midbody, tail) and conditions (swimming, crawling, day 1 adults, day 5 adults). For muscle atrophy analysis, similar measurements were compared between wild-type and *dys-1 (eg33)* worms.

### Statistical Analysis

Statistical analyses were performed using SigmaPlot 14.5. Data normality was assessed using the Shapiro-Wilk test, and equal variance was evaluated with the Brown-Forsythe test. Outliers were identified using the interquartile range method and excluded from the analysis. For comparisons of three body regions, One-Way ANOVA was conducted, followed by Holm-Sidak post hoc tests. Non-parametric data were analyzed using the Kruskal-Wallis One Way Analysis of Variance on Ranks, followed by Dunn’s post hoc tests. Statistical significance was defined at an alpha of 0.05. A Bonferroni correction was applied to the comparisons involving day 1 crawling worms, where the data for allometric growth, developmental growth, and hypertrophic growth were compared. The original alpha of 0.05 was divided by the number of comparisons being made. In this case, the corrected alpha was set to 0.017 (0.05/3). Additionally, the Bonferroni correction was used when comparing day 5 crawling worms for developmental growth and atrophy. In this case where two comparisons were made, the corrected alpha was set to 0.025 (0.05/2). Statistical power (1-β) was maintained at 0.80. For non-parametric tests, statistical power was estimated using the Monte Carlo Simulation method. Detailed descriptive and comparative statistics for each figure, along with raw data, are provided in the supplementary files.

## Figures and Tables

**Table 1.** Pearson product moment correlation analysis between 26 myocytes ranging in area between 561μm^2^ and 1,738μm^2^ measured using ImageJ on confocal images of day 1 adult *C. elegans* raised on agar plates. Values correspond to N>5 per myocyte. Pearson Coefficients (PC) and p values are reported. The alpha was α=0.05 and power of the comparison was 1-β=0.96.

	Muscle area	Sarcomere width	A-band width	I-band width	Sarcomere number
**Myocyte area**		PC= 0.43 p= 0.023	PC= 0.52 p=0.006	PC= 0.51 p=0.008	PC= 0.60 p= 0.001
**Sarcomere width**	PC= 0.43 p= 0.023		PC= 0.59 p= 0.001	PC= 0.54 p= 0.02	PC= 0.10 p= 0.63
**A-band width**	PC= 0.52 p=0.006	PC= 0.59 p= 0.001		PC= 0.07 p= 0.75	PC= 0.43 p= 0.03
**I-band width**	PC= 0.51 p=0.008	PC= 0.54 p= 0.02	PC= 0.07 p= 0.75		PC= 0.04 p= 0.83
**Sarcomere number**	PC= 0.60 p= 0.001	PC= 0.10 p= 0.63	PC= 0.43 p= 0.03	PC= 0.04 p= 0.83	

## References

[R1] Ajayi P.T., Katti P., Zhang Y., Willingham T.B., Sun Y., Bleck C.K.E. and Glancy B. (2022). Regulation of the evolutionarily conserved muscle myofibrillar matrix by cell type dependent and independent mechanisms. Nature Communications, 13(1). doi:10.1038/s41467-022-30401-9. PMC9106682 35562354

[R2] Beron C, Vidal‐Gadea AG, Cohn J, Parikh A, Hwang G, Pierce‐Shimomura JT. The burrowing behavior of the nematode Caenorhabditis elegans: a new assay for the study of neuromuscular disorders. Genes, Brain and Behavior. 2015 Apr;14(4):357–68. 25868909 10.1111/gbb.12217 PMC4444045

[R3] Blake D. J., Weir A., Newey S. E., & Davies K. E. (2002). Function and genetics of dystrophin and dystrophin-related proteins in muscle. Physiological Reviews, 82(2), 291–329. [DOI: 10.1152/physrev.00028.2001](10.1152/physrev.00028.2001) 11917091

[R4] Brenner S. (1974). The Genetics of Caenorhabditis elegans. Genetics, [online] 77(1), pp.71–94. doi:10.1093/genetics/77.1.71. 4366476 PMC1213120

[R5] Ellwood RA, Piasecki M, Szewczyk NJ. Caenorhabditis elegans as a model system for duchenne muscular dystrophy. International Journal of Molecular Sciences. 2021 May 5;22(9):4891. 34063069 10.3390/ijms22094891 PMC8125261

[R6] Fazyl A, Sawilchik E, Stein W, Vidal-Gadea AG. Muscular expression of pezo-1 differentially contributes to swimming and crawling production in the nematode C. elegans. bioRxiv. 2024:2024–08.

[R7] Frontera WR, Ochala J. Skeletal muscle: a brief review of structure and function. Calcified tissue international. 2015 Mar;96:183–95. 25294644 10.1007/s00223-014-9915-y

[R8] Gieseler K, Qadota H, Benian GM. Development, structure, and maintenance of C. elegans body wall muscle. WormBook: the online review of C. elegans biology [Internet]. 2018. 10.1895/wormbook.1.81.2 PMC5410635 27555356

[R9] Glass D. J. (2005). Skeletal muscle hypertrophy and atrophy signaling pathways. Nature Reviews Molecular Cell Biology, 6(1), 42–50. [DOI: 0.1016/j.biocel.2005.04.018) 10.1016/j.biocel.2005.04.018 16087388

[R10] Goldspink G. Sarcomere length during post-natal growth of mammalian muscle fibres. Journal of cell science. 1968 Dec 1;3(4):539–48. 5707851 10.1242/jcs.3.4.539

[R11] Højfeldt G., Sorenson T., Gonzales A., Kjaer M., Andersen J.L. and Mackey A.L. (2023). Fusion of myofibre branches is a physiological feature of healthy human skeletal muscle regeneration. Skeletal muscle, 13(1). doi:10.1186/s13395-023-00322-2. PMC10422711 37573332

[R12] Hoffman E. P., Brown R. H., & Kunkel L. M. (1987). Dystrophin: The protein product of the Duchenne muscular dystrophy locus. Cell, 51(6), 919–928. [DOI: 10.1016/0092-8674(87)90579-4](10.1016/0092-8674(87)90579-4 ) 3319190

[R13] Hughes KJ, Rodriguez A, Flatt KM, Ray S, Schuler A, Rodemoyer B, Veerappan V, Cuciarone K, Kullman A, Lim C, Gutta N. Physical exertion exacerbates decline in the musculature of an animal model of Duchenne muscular dystrophy. Proceedings of the National Academy of Sciences. 2019 Feb 26;116(9):3508–17. 10.1073/pnas.1811379116 PMC6397527 30755520

[R14] Mendoza E, Moen DS, Holt NC. The importance of comparative physiology: mechanisms, diversity and adaptation in skeletal muscle physiology and mechanics. Journal of Experimental Biology. 2023 Apr 25;226(Suppl_1):jeb245158. 36960844 10.1242/jeb.245158

[R15] Moerman D. G., & Fire A. (1997). Muscle: Structure, function, and development. In C. elegans II (pp. 417–470). Cold Spring Harbor Laboratory Press. 21413235

[R16] Moerman DG, Williams BD. Sarcomere assembly in C. elegans muscle. WormBook: The Online Review of C. elegans Biology [Internet]. 2006 Jan 16. 10.1895/wormbook.1.81.1 PMC4781162 18050483

[R17] Pierce-Shimomura JT, Chen BL, Mun JJ, Ho R, Sarkis R, McIntire SL. Genetic analysis of crawling and swimming locomotory patterns in C. elegans. Proceedings of the National Academy of Sciences. 2008 Dec 30;105(52):20982–7. 10.1073/pnas.0810359105 PMC2634943 19074276

[R18] Sartori R, Romanello V, Sandri M. Mechanisms of muscle atrophy and hypertrophy: implications in health and disease. Nature communications. 2021 Jan 12;12(1):330. 10.1038/s41467-020-20123-1 PMC7803748 33436614

[R19] Schiaffino S, Dyar KA, Ciciliot S, Blaauw B, Sandri M. Mechanisms regulating skeletal muscle growth and atrophy. The FEBS journal. 2013 Sep;280(17):4294–314. 23517348 10.1111/febs.12253

[R20] Schiaffino S., & Mammucari C. (2011). Regulation of skeletal muscle growth by the IGF1-Akt/PKB pathway: insights from genetic models. Trends in Pharmacological Sciences, 32(11), 473–481. 10.1186/2044-5040-1-4 PMC3143906 21798082

[R21] Schiaffino S., & Reggiani C. (2011). Fiber types in mammalian skeletal muscles. Physiological Reviews, 91(4), 1447–1531. [DOI: 10.1152/physrev.00031.2010]. 22013216

[R22] Schoenfeld BJ. The mechanisms of muscle hypertrophy and their application to resistance training. The Journal of Strength & Conditioning Research. 2010 Oct 1;24(10):2857–72. 20847704 10.1519/JSC.0b013e3181e840f3

[R23] Stiernagle T. Maintenance of C. elegans. WormBook: The online review of C. elegans biology [Internet]. 2006 Feb 11. 10.1895/wormbook.1.101.1 PMC4781397 18050451

[R24] Vidal-Gadea A, Topper S, Young L, Crisp A, Kressin L, Elbel E, Maples T, Brauner M, Erbguth K, Axelrod A, Gottschalk A. Caenorhabditis elegans selects distinct crawling and swimming gaits via dopamine and serotonin. Proceedings of the National Academy of Sciences. 2011 Oct 18;108(42):17504–9. 10.1073/pnas.1108673108 PMC3198358 21969584

[R25] White R.B., Biérinx A.-S., Gnocchi V.F. and Zammit P.S. (2010). Dynamics of muscle fibre growth during postnatal mouse development. BMC Developmental Biology, 10(1). doi: 10.1186/1471-213x-10-21. PMC2836990 20175910

[R26] Willingham TB, Kim Y, Lindberg E, Bleck CK, Glancy B. The unified myofibrillar matrix for force generation in muscle. Nature communications. 2020 Jul 24;11(1):3722. 10.1038/s41467-020-17579-6 PMC7381600 32709902

[R27] Wokke BH, Van Den Bergen JC, Versluis MJ, Niks EH, Milles J, Webb AG, Van Zwet EW, Aartsma-Rus A, Verschuuren JJ, Kan HE. Quantitative MRI and strength measurements in the assessment of muscle quality in Duchenne muscular dystrophy. Neuromuscular Disorders. 2014 May 1;24(5):409–16. 24613733 10.1016/j.nmd.2014.01.015

