# Activity-Dependent Sarcomere Remodeling in *C. elegans* Muscle Correlates with Mechanical Vulnerability in Dystrophy

**DOI:** 10.1101/2024.08.30.610496

**Published:** 2025-05-14

**Authors:** A Fazyl, A Anbu, S Kollbaum, E Conklin, N Schroeder, AG Vidal-Gadea

**Affiliations:** 1 School of Biological Sciences, Illinois State University, Normal, IL; 2 Department of Crop Sciences, University of Illinois, Urbana, IL

## Abstract

Sarcomere networks (formed by lateral connections between myofibrils) are essential for force distribution in striated muscle. Yet, whether these networks remodel to meet changing mechanical demands or contribute to muscle pathology is unclear. Using *C. elegans* body-wall muscles, we show that sarcomeres form interconnected networks via actin-, myosin-, and actinin-rich junctions that dynamically remodel in response to locomotor activity. Swimming, which imposes greater mechanical demands, enhances sarcomere branching compared to crawling. In dystrophin-deficient *dys-1(eg33)* mutants, impaired longitudinal anchorage combined with persistent lateral tension leads to myofibrillar buckling and wavy fiber deformation, echoing features of Duchenne muscular dystrophy. These results position *C. elegans* as a tractable model for dissecting the biomechanical roles of sarcomere networks in muscle adaptation and disease.

## Introduction

Striated muscles generate force through sarcomeres organized into myofibrils. While classically depicted as parallel cylinders, recent 3D reconstructions reveal interconnected sarcomere networks that distribute force laterally in vertebrates (1, 2) and select invertebrate muscles, such as Drosophila tubular muscles (3). A key pathological feature of Duchenne muscular dystrophy (DMD) is wavy, undulating muscle fibers, suggesting disrupted mechanical organization (4). However, whether sarcomere networks are dynamically regulated by mechanical activity (or contribute to pathology when structurally compromised) remains unknown.

The nematode *C. elegans*, with its genetically tractable body-wall muscles, offers an ideal system to address these questions (5–9). Unlike *Drosophila*, where sarcomere branching is genetically regulated by transcription factors (e.g., salm, HIS) or cytoskeletal proteins (e.g., neurochondrin) (3), *C. elegans* enables direct interrogation of activity-dependent plasticity due to its ability to perform multiple distinct and quantifiable locomotor behaviors like crawling and swimming (10–12). Here, we demonstrate that *C. elegans* muscles form dynamic sarcomere networks that remodel with mechanical demand and exhibit structural failure in dystrophin-deficient mutants, mirroring DMD pathology.

## Results

### Sarcomere structure visualized and quantified in individual body-wall myocytes.

**Figure 1.**
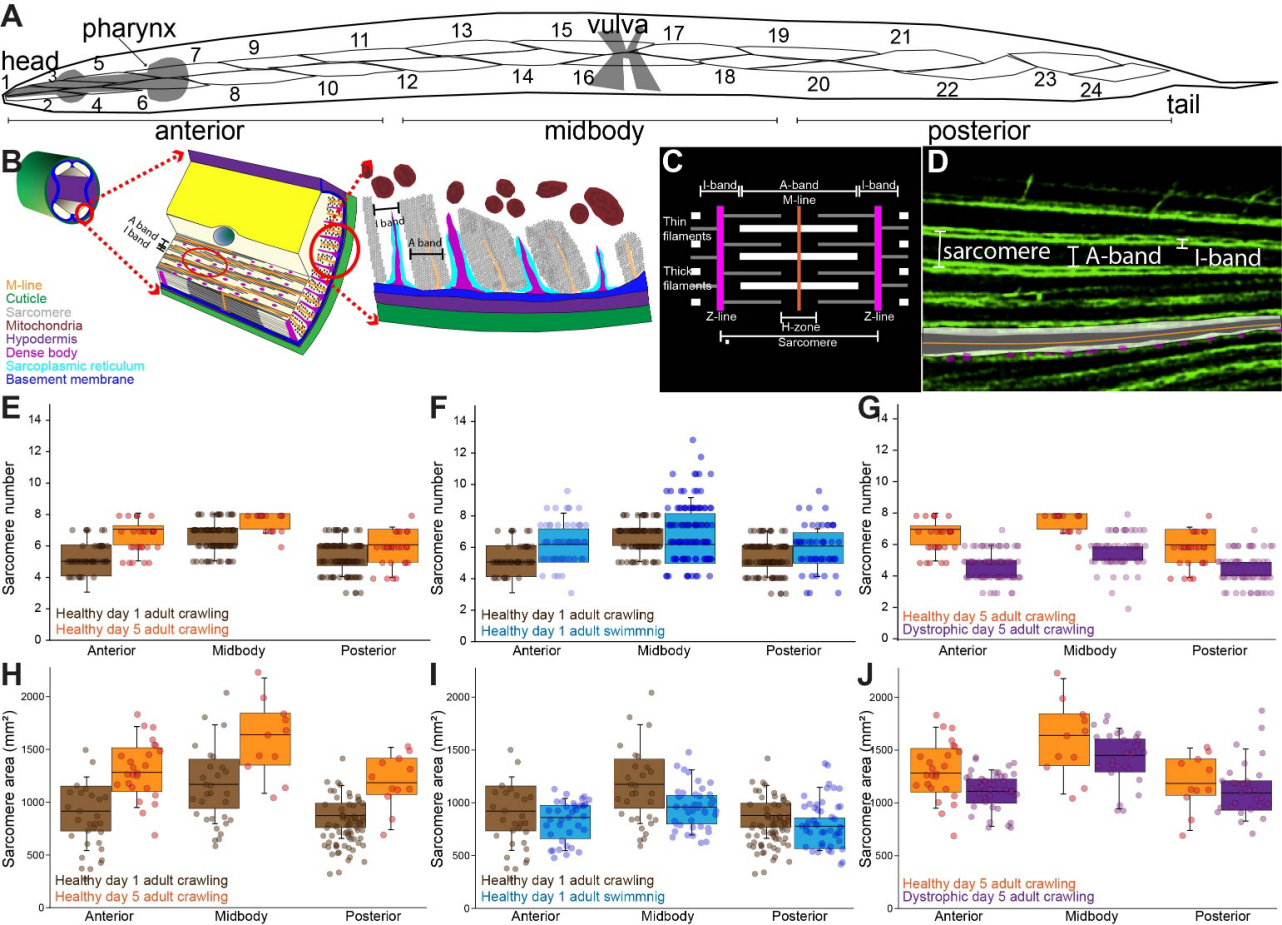
Sarcomere number and area are modulated by body region, developmental stage, locomotor behavior, and dystrophy. (A) Schematic of *C. elegans* showing anterior, medial, and posterior muscle segments analyzed. (B) Schematic cross-section of a body-wall myocyte illustrating parallel myofibrils (after Gieseler et al., 2018). (C) Diagram of a single sarcomere bordered by Z-lines (magenta), showing I-, A-, and H-band sub-regions. (D) Lightning deconvolved confocal micrograph of a phalloidin-stained day-1 adult muscle (phalloidin labels actin), illustrating the landmarks defined in (A,B). (E–G) Sarcomere number per myocyte across experimental conditions. (E) Healthy animals show increased sarcomere number with age and significant regional differences (aligned rank transform (ART) ANOVA main effects of region and age). (F) Dystrophic muscles display reduced sarcomere number and altered regional scaling in crawling animals (main effects of region and health; significant region × health interaction). (G) Day-1 healthy animals show increased sarcomere number during swimming compared to crawling, in a region-specific manner (significant region × locomotion interaction). (H–J) Sarcomere area per myocyte under the same comparisons. (H) Healthy animals show increased sarcomere area with age and regional variation (main effects only). (I) Dystrophic animals show reduced sarcomere area with strong regional differences (main effects; no interaction). (J) Swimming increases sarcomere area compared to crawling across all regions (main effects of region and locomotion). Each point represents a single myocyte measurement. Boxplots show median, interquartile range (IQR), and 1.5× IQR whiskers; individual data points are overlaid. Statistical comparisons were performed using ART ANOVA. Significance levels: p < 0.05; p < 0.01; *p < 0.001.

To anchor the quantitative analyses that follow, Figure 1 combines a schematic of *C. elegans* muscle segments (Figure 1A), a schematic cross-section of a body-wall myocyte (Figure 1B, adapted from Gieseler *et al.*, 2018), and a diagram of sarcomere subregions (Figure 1C). The phalloidin-stained image in Figure 1D shows actin-rich sarcomeric structures in a single body-wall myocyte, establishing the sarcomere-associated area and number used throughout the study. All measurements were based on planar 2D projections and do not reflect volumetric muscle size.

### Regional and developmental variation in sarcomere number and area.

To assess how sarcomeric structure varies across the body, we quantified sarcomere number and area in actin-stained myocytes from anterior (myocytes 1–10), medial (11–18), and posterior (19–25) regions. We performed three aligned rank transform (ART) ANOVAs on sarcomere number and three on sarcomere area to test the effects of body region, developmental stage (day 1 vs. day 5 adults), locomotor condition (crawling vs. swimming), and dystrophy.

In healthy animals, both sarcomere number and area increased significantly with age and varied by body region (Figure 1E,H). ART ANOVA revealed a main effect of age (F_1,361_ = 11.38, p = 0.0008 for number; F_1,301_ = 71.28, p < 0.0001 for area) and region (F_2,361_ = 3.30, p = 0.038 for number; F_2,301_ = 6.62, p = 0.0015 for area), with no significant interactions, indicating uniform growth across body regions.

### Locomotor activity drives region-specific remodeling of sarcomeric architecture.

To determine whether sarcomeric remodeling differs with locomotor mode, we compared day 1 healthy animals reared under standard crawling or swimming conditions. Swimming significantly modulated sarcomere structure in a region-dependent manner: ART ANOVA revealed a significant region × locomotion interaction for sarcomere number (F_2,299_ = 9.43, p < 0.0001), with medial muscles showing the largest increases (Figure 1G). Sarcomere area also increased with swimming (F_1,253_ = 7.40, p = 0.007), though the effect was consistent across regions (Figure 1J).

### Dystrophy alters sarcomere morphology and regional scaling.

We next examined the impact of dystrophy. In crawling animals, ART ANOVA detected significant effects of dystrophy on both sarcomere number (F_1,220_ = 14.55, p = 0.0002) and area (F_1,272_ = 16.52, p = 0.00006), with dystrophic animals showing marked reductions. Regional effects were also significant for number (F_2,220_ = 12.02, p < 0.0001) and area (F_2,272_ = 33.78, p < 0.0001), and there was a significant region × health interaction for sarcomere number (F_2,220_ = 3.08, p = 0.048; Figure 1F,I), suggesting enhanced vulnerability in specific muscle groups.

### Sarcomere networks form via branching and splitting.

**Figure 2.**
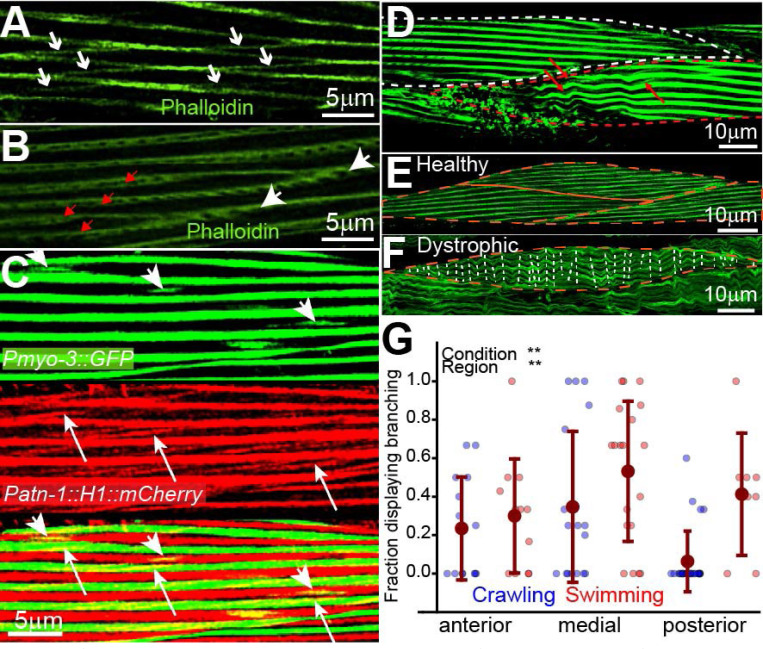
Sarcomere network remodeling and structural defects in dystrophic *C. elegans* muscle. (A) Confocal micrograph showing sarcomere branching in wildtype body-wall muscle. (B) High-magnification confocal image showing sarcomere splitting (white arrows: parent sarcomeres; red arrows: newly formed branches). (C) Dual fluorescence images of body-wall muscle in animals co-expressing *Pmyo-3::GFP* (myosin, green) and *Patn-1::mCherry* (actinin, red) to show colocalization of contractile and structural proteins: (i) *Pmyo-3::GFP*, (ii) *Patn-1::mCherry*, (iii) merged image. (D) Confocal image of dystrophic *dys-1(eg33)* muscle showing detached sarcomeres and fiber damage. Characteristic wavy pattern coincides with sarcomere branch attachment sites (red arrows). (E) Healthy wildtype body-wall myocyte showing straight, organized sarcomeres. (F) *dys-1(eg33)* dystrophic myocyte showing wave-like deformation and wavy fibers with detachment from neighboring muscles (dashed white lines indicate individual waves). (G) Quantification of sarcomere branching frequency across locomotor conditions and body regions; swimming animals display significantly increased branching relative to crawling animals (aligned rank transform (ART) ANOVA main effect of condition, p < 0.001; main effect of region, p = 0.0016; no interaction, p = 0.231). Points represent individual muscle measurements, colored by locomotor condition (blue = crawling, red = swimming). Data shown as mean ± SD.

High-resolution confocal imaging revealed lateral actin-rich interconnections consistent with sarcomere branching (Figure 2A), and Y-shaped bifurcations indicative of sarcomere splitting (Figure 2B). To determine whether these features contained contractile machinery, we examined a dual-reporter strain expressing fluorescently tagged myosin and actinin. Both thick filaments (myosin) and dense body proteins (actinin) colocalized with actin at branch points and along bifurcating sarcomeres (Figure 2C), indicating that these structures contain core contractile components. These observations were further supported by transmission electron microscopy: serial EM sections revealed sarcomere branching and splitting events in which thin and thick filaments, as well as dense body–like electron-dense structures (Z-disks), were present throughout the bifurcation (Supplementary Video 1). Together, these data confirm that sarcomere branches are not merely structural extensions but are likely contractile units integrated into the force-generating network.

### Dystrophic fibers display wave-like deformation due to failed anchorage.

In dystrophic dys-1(eg33) mutants, actin filaments formed sinusoidal waves instead of linear bundles (Figure 2D–F), consistent with branch-mediated bending in the absence of stable end anchorage. These wavy fiber phenotypes resemble pathological features in human Duchenne muscular dystrophy (4).

### Sarcomere branching is activity-dependent.

To test whether sarcomere branching is plastic, we quantified the fraction of myocytes exhibiting branching under different conditions and analyzed the data using ART ANOVA. Branching frequency was significantly higher in swimming than crawling animals (F_1,91_ = 16.51, p < 0.001) and varied by region (F_2,91_ = 6.94, p = 0.0016), but the region × condition interaction was not significant (F_2,91_ = 1.49, p = 0.23; Figure 2G). A direct comparison between day 1 and day 5 crawling animals using ART ANOVA showed no statistically significant difference (F_1,39_ = 1.83, p = 0.18), indicating that locomotor activity rather than age is the primary driver of remodeling. These findings support the hypothesis that sarcomere networks dynamically reorganize in response to mechanical demands.

### Branching quantification in dystrophic muscles was inconclusive.

We attempted to quantify sarcomere branching in *dys-1(eg33)* animals using the same imaging and analysis pipeline applied to wild-type worms. However, the wavy deformation of actin filaments in dystrophic muscles made it challenging to distinguish true sarcomere branches from structural distortions. Since the compromised architecture prevented reliable quantification, we cannot determine whether sarcomere network remodeling is altered in dystrophic animals.

## Discussion

Recent studies in vertebrate muscle have revealed that sarcomeres are not strictly linear elements but instead form highly interconnected, branching networks critical for lateral force distribution (1, 2). Here, we show that *C. elegans* striated muscles similarly harbor sarcomere networks that include branching and splitting events, some of which contain thick filaments (myosin), dense bodies (actinin), and thin filaments. These contractile components were observed to colocalize at bifurcations in both dual-reporter strains and serial electron micrographs, suggesting that sarcomere branches in *C. elegans* are structurally capable of force generation.

Our quantitative data further indicate that these networks are dynamically modulated by mechanical context. Specifically, we observed significant increases in sarcomere number, area, and branching in animals subjected to swimming versus crawling, consistent with activity-dependent remodeling. In contrast, aging (day 5 vs. day 1) did not significantly alter branching, pointing to mechanical activity as the key regulatory driver.

In dystrophin-deficient animals, we observed wavy, sinusoidal myofibrils aligned with branch points, consistent with models where intact lateral tension (unopposed by anchorage) leads to buckling. However, our attempts to quantify sarcomere branching in dystrophic fibers were inconclusive due to severe architectural disruption, preventing resolution of whether network remodeling is preserved or compromised under these conditions.

Altogether, our findings suggest that *C. elegan*s possesses a dynamic sarcomere network architecture that parallels vertebrate myofibrillar matrices in both structure and regulation. This conserved feature of muscle organization offers a genetically tractable platform for dissecting the biomechanical basis of sarcomere remodeling in health and disease.

## Materials and Methods

### Strains and maintenance.

*C. elegans* were reared at 20°C on nematode-growth-medium (NGM) agar seeded with *E. coli* OP50 (1). Strains: wild-type N2, dystrophic BZ33 *dys-1(eg33)*, and cross of DM8005 [*myo-3p::GFP::myo-3* + *rol-6(su1006)*] for GFP–myosin and RW11481 [*atn-1::H1-wCherry*] for mCherry–actinin.

### Animals used.

The data presented in this study was derived from Day 1 crawling N2 (WT) animals (12 animals for 39 anterior myocytes, 12 animals for 37 midbody myocytes, and 9 animals for 25 posterior myocytes), Day 1 swimming N2 (WT) animals (12 animals for 45 anterior myocytes, 19 animals for 82 midbody myocytes, and 8 animals for 24 posterior myocytes), Day 5 N2 (WT) animals (14 animals for 54 anterior myocytes, 9 animals for 32 midbody myocytes, and 6 animals for 22 posterior myocytes), and Day 5 BZ33 (dystrophic) animals (15 animals for 76 anterior myocytes, 15 animals for 61 midbody myocytes, and 15 animals for 64 posterior myocytes). Please see Supplementary table 1 for measurements. Raw data used in this project can be accessed through the following link: https://figshare.com/s/8ea23d743d7c3e5740d0.

### Experimental design.

Synchronized embryos were obtained by alkaline hypochlorite bleaching (2) and placed either on hard NGM plates (crawling regimen) or in liquid NGM in eppendorf tubes (swimming regimen). Animals were analyzed as day-1 adults or day-5 adults. Anterior, mid-body, and posterior myocytes correspond to myocytes 1–10, 11–18, and 19–25, respectively.

### Fixation and staining.

Animals at the desired stages (day 1 and day 5 adults) were collected from the plates or liquid media and fixed in 4% paraformaldehyde in phosphate-buffered saline for 15 mins at room temperature. Fixed worms were then subjected to freeze-cracking on poly-L-lysine slides to permeabilize the cuticle (3). Following permeabilization, samples were stained with iFluor 488-conjugated phalloidin overnight to visualize F-actin filaments.

### Confocal imaging.

Images were acquired on a Leica SP8 with Lightning deconvolution (63×, NA 1.40 OIL). Z-stacks were captured, with the number and size of each Z-step optimized for each myocyte to properly capture the structure along the depth for subsequent deconvolution (typically ~15–30 slices with Z-steps ~0.17μm). Raw image stacks were processed using Leica’s Lightning deconvolution algorithm within Leica Application Suite X (LAS X, v3.5.5.19976) software. For each myocyte we used the phalloidin staining F-actin of the sarcomeres to count sarcomere number, and we then measured sarcomere area as the outline traced around stained sarcomeres from maximum projection micrographs. Analyses used LAS X and Fiji/ImageJ.

### Electron microscopy.

Archived digitized electron microscopy prints (Specimen N2T print # 300–848), originally collected by Nichol Thomson at the Medical Research Council in Cambridge, UK and now housed at the Center for *C. elegans* Anatomy, were aligned using TrakEM2 (Cardona *et al.*, 2012). Access to image files is available at www.wormimage.org. Any images where the muscle was obscured were excluded. Aligned images were exported as an AVI file and edited using Adobe Premiere Pro.

### Statistics.

Statistical analyses were conducted using R (version 4.4.3) and the ARTool package (4, 5), which implements aligned rank transform (ART) ANOVA for non-parametric factorial designs. This approach allows testing of main effects and interactions in data that violate normality or homoscedasticity assumptions.

To assess how sarcomere number and area varied with body region, age, locomotor behavior, and health status, we ran three separate ART ANOVAs for each dependent variable. First, we analyzed healthy animals to examine effects of region × age and region × locomotion. Second, to test the effects of dystrophy, we analyzed crawling-only animals, comparing healthy and dystrophic groups across regions. To ensure valid statistical comparisons, subsets with incomplete factorial combinations (e.g., no dystrophic swimmers or day 5 swimmers) were excluded from specific models, and all models used Type III sums of squares. A significance threshold of α = 0.05 was used for all ART ANOVAs.

To estimate the sensitivity of our statistical tests, we back-calculated approximate effect sizes (η² and Cohen’s *d* or *f*) from observed F-values and degrees of freedom. The interaction between region and locomotor behavior on sarcomere number (F(2,299) = 9.43) yielded a medium effect (Cohen’s *f* ≈ 0.25), while the main effect of dystrophy on sarcomere area (F(1,272) = 16.52) corresponded to Cohen’s *d* ≈ 0.49 (medium). The effect of locomotor condition on sarcomere branching frequency (F(1,91) = 16.51) produced a large effect (Cohen’s *d* ≈ 0.85). Given our group sizes, these effects were detected with an estimated power exceeding 80–95%, indicating that our study was well-powered to identify moderate-to-large effects.

## Supplementary Material

Supplement 1

## Data Availability

Data used in this study is accessible through the following link: https://figshare.com/s/8ea23d743d7c3e5740d0.

## Significance Statement

Sarcomere networks interconnect contractile fibers and are thought to distribute forces laterally across muscles. However, whether these networks dynamically remodel to meet mechanical demands or contribute to disease remains unknown. Here, we show that sarcomere networks in *C. elegans* body-wall muscles display activity-dependent plasticity and incorporate force-generating components. In dystrophin-deficient mutants, the persistence of lateral tension, combined with impaired longitudinal anchorage, correlates with myofibrillar buckling and wavy fiber deformation. These findings suggest that sarcomere networks are dynamic mechanical structures that adapt to contractile demands and influence muscle integrity during disease

## References

[1] Højfeldt G., , Fusion of myofibre branches is a physiological feature of healthy human skeletal muscle regeneration. Skeletal Muscle 13, 13 (2023). 37573332 10.1186/s13395-023-00322-2 PMC10422711

[2] Willingham T. B., Kim Y., Lindberg E., Bleck C. K. E., Glancy B., The unified myofibrillar matrix for force generation in muscle. Nat Commun 11, 3722 (2020). 32709902 10.1038/s41467-020-17579-6 PMC7381600

[3] Ajayi P. T., , Regulation of the evolutionarily conserved muscle myofibrillar matrix by cell type dependent and independent mechanisms. Nat Commun 13, 2661 (2022). 35562354 10.1038/s41467-022-30401-9 PMC9106682

[4] Tyler K. L., Origins and early descriptions of “Duchenne muscular dystrophy.” Muscle and Nerve 28, 402–422 (2003). 14506712 10.1002/mus.10435

[5] Moerman D. G., Fire A., Riddle D. I., elegans II C.. (1997).

[6] Pierce-Shimomura J. T., , Genetic analysis of crawling and swimming locomotory patterns in C. elegans. Proc. Natl. Acad. Sci. U.S.A. 105, 20982–20987 (2008). 19074276 10.1073/pnas.0810359105 PMC2634943

[7] Petzold B. C., , Caenorhabditis elegans Body Mechanics Are Regulated by Body Wall Muscle Tone. Biophysical Journal 100, 1977–1985 (2011). 21504734 10.1016/j.bpj.2011.02.035 PMC3077690

[8] Gieseler K., Development, structure, and maintenance of C. elegans body wall muscle. WormBook 1–59 (2017). 10.1895/wormbook.1.81.2. PMC5410635 27555356

[9] Ellwood R. A., Piasecki M., Szewczyk N. J., Caenorhabditis elegans as a Model System for Duchenne Muscular Dystrophy. IJMS 22, 4891 (2021). 34063069 10.3390/ijms22094891 PMC8125261

[10] FazylA., , Muscular expression of pezo-1 differentially influences swimming and crawling in C. elegans. Biophysical Journal S0006349524041262 (2024). 10.1016/j.bpj.2024.12.032.41429758

[11] Laranjeiro R., , Swim exercise in Caenorhabditis elegans extends neuromuscular and gut healthspan, enhances learning ability, and protects against neurodegeneration. Proc. Natl. Acad. Sci. U.S.A. 116, 23829–23839 (2019). 31685639 10.1073/pnas.1909210116 PMC6876156

[12] Vidal-Gadea A., , Caenorhabditis elegans selects distinct crawling and swimming gaits via dopamine and serotonin. Proceedings of the National Academy of Sciences 108, 17504–17509 (2011). 10.1073/pnas.1108673108 PMC3198358 21969584

