# Supplementary material for: Activity-Dependent Sarcomere Remodeling in C. elegans Muscle Correlates with Mechanical Vulnerability in Dystrophy
Source: bioRxiv. 2025 May 14:2024.08.30.610496. Originally published 2024 Aug 30. Preprint. [Version 2] doi: 10.1101/2024.08.30.610496 (PMC11383985; doi:10.1101/2024.08.30.610496)
Supplement: Supplement 1 [file NIHPP2024.08.30.610496v2-supplement-1.pdf]

bioRxiv preprint doi: https://doi.org/10.1101/2024.08.30.610496; this version posted May 14, 2025. The copyright holder for this preprint (which was not certified by peer review) is the author/funder, who has granted bioRxiv a license to display the preprint in perpetuity. It is made available under aCC-BY 4.0 International license.

Supplementary captions

Supplementary Video 1. Serial electron micrographs reveal sarcomere splitting and structural continuity in *C. elegans* body-wall muscle. Electron microscopy images of a *C. elegans* adult (N2T dataset collected from Medical Research Council and currently housed at Center for *C. elegans* Anatomy) were digitized. Then, individual images were aligned using TrakEM2 in ImageJ (6). After alignment, images were exported and then

bioRxiv preprint doi: https://doi.org/10.1101/2024.08.30.610496; this version posted May 14, 2025. The copyright holder for this preprint (which was not certified by peer review) is the author/funder, who has granted bioRxiv a license to display the preprint in perpetuity. It is made available under aCC-BY 4.0 International license.

reimported into ImageJ where they were formatted into an avi video file. Then, using Adobe Premiere Pro, the video was edited to contain text and segmented sections in order to aid understanding.

References

1. S. Brenner, the genetics of *Caenorhabditis elegans*. *Genetics* **77**, 71–94 (1974).
2. T. Stiernagle, Maintenance of *C. elegans*. *WormBook* (2006). https://doi.org/10.1895/wormbook.1.101.1.
3. J. S. Duerr, Antibody Staining in *C. elegans* Using Freeze-Cracking; *JoVE* 50664 (2013). https://doi.org/10.3791/50664-v.
4. J. O. Wobbrock, L. Findlater, D. Gergle, J. J. Higgins, The aligned rank transform for nonparametric factorial analyses using only anova procedures in *Proceedings of the SIGCHI Conference on Human Factors in Computing Systems*, (ACM, 2011), pp. 143–146.
5. L. A. Elkin, M. Kay, J. J. Higgins, J. O. Wobbrock, An Aligned Rank Transform Procedure for Multifactor Contrast Tests in *The 34th Annual ACM Symposium on User Interface Software and Technology*, (ACM, 2021), pp. 754–768.
6. A. Cardona, *et al.*, TrakEM2 Software for Neural Circuit Reconstruction. *PLoS ONE* **7**, e38011 (2012).
